# 

**Published:** 1840-01-01

**Authors:** 


### THE MEDICO-CHIRURGICAL REVIEW,

AND

### JOURNAL

OF

### PRACTICAL MEDICINE.

(*NEW SERIES.*)

VOLUME THIRTY-TWO,

[1st of OCTOBER, 1839, to 31st of MARCH,]

1840.

VOL. XII. of DECENNIAL SERIES.

EDITED

BY JAMES JOHNSON, M.D.

PHYSICIAN EXTRAORDINARY TO THE LATE KING,

AND

HENRY JAMES JOHNSON, ESQ.

LECTURER ON ANATOMY AT THE SCHOOL OF ST. GEORGE'S HOSPITAL IN KINNERTON STREET.

Surgeon Gen'l's Office LIBRARY. 57472 Washington, D.C.

LONDON:

PUBLISHED BY S. HIGHLEY, 32, FLEET STREET,

*Re-printed in New York, by Mr. Wood.*

1840.

W1
ME 75 T

PRINTED BY F. HAYDEN,
Little College Street, Westminster.

### INDEX.

A.

Abdomen, pathology of the veins in the 524
Abdominal parietes, sloughing of .... 262
Absence of tears in infants ........ 385
Absence of the vagina, complete .... 531
Abscess between the pharynx and spine 582
Acetate of lead in aneurysm of the aorta ................ ....... 210
Acetate of lead for cholera ........ 593
Aconite, poisoning from the extract of 239
Acute rheumatism, Dr. Corrigan on.. 266
Acute glanders ................. 543
Addison's practice of medicine...... 136
Advantages of delay in comminuted fractures ...................... 216
Ague .... ..................... 137
Alcock, Mr. ..................... 601
Alderson on the effects of lead upon the system ..................... 11
Algiers, diseases prevalent at........ 516
Alterations of the blood, Magendie on 228
Amputation, Mr. Bransby Cooper on 257
Amputation of the penis .......... 532
Amsterdam lunatic asylum ........ 565
Anasarcous tumour of the scrotum, Liston on . .................. 19
Anatomical text-book ............ 167
Anatomical subjects, preservation of 248
Anatomists manual, by Maygrier .... 167
Anatomy and Physiology, Cyclopædia of............................ 81
Anatomy of the groin, Morton's .... 167
Anatomy of the arteries, Flood's .... 167
Anatomy of the breast, by Cooper .. 360
Aneurysm of the aorta, acetate of lead in........................... 210
Aneurysm cured by the needle...... 280
Aneurysm, brachial, sloughing of a.. 281
Aneurysm by anastomosis.......... 607
Aneurysm of the internal carotid. .. 575
Aneurysm of the brachial artery, Mr. H. Johnson's case of ............ 599
Aneurysmal tumor of the orbit .... 22
Aneurysms........................ 410
Animal heat ................... 95
Animal heat, relations of age to .... 105
Animal heat, influence of seasons on 109
Animal kingdom, Mr. Rymer Jones's 192
Annual reports of poor-law commissioners .................... 113
Aorta, rupture of the............... 612
Aphthæ ......................... 390
Aphthous inflammation of the mouth 533
Apoplexy of the pons varolii ...... 33
Apoplexy, treatment of ............ 545
Appendix cæci, inflammation of the.. 46
Appendix cæci, perforation of the .. 46
Aqua-membranitis................ 458
Arachnitis ...................... 146
Areola, of the.................... 366
Arm, gun-shot wound of the ...... 608
Arnott's case of malignant disease of the tongue ...................... 14
Arsenic, poisoning by ............ 220
Arsenic, antidotes of .............. 225
Arsenic, detection of, in the blood .. 226
Arsenic, new combination of........ 279
Arsenic in the bones .............. 542
Arteries, Flood's surgical anatomy of 167
Arteries of the limbs, alterations peculiar to ......................... 465
Arteritis ......................... 460
Artificial anus, operation for ...... 539
Asphyxia of new-born infants...... 406
Assistant-surgeons in the navy, regulations respecting ................. 601
Asylum at Utrecht................ 565
Athens, medical school at ......... 275
Attitudes of the child .......... . 382
Auscultation ..................... 254
Axillary arteries, rupture of the .... 267

B.

Banks on medical etiquette ........ 62
Barbarous operation for phymosis .. 156
Beddoes, anecdote of .............. 326
Bell's history of British reptiles .... 192
Bilious diarrhœa ................ 399
Billard on the diseases of infants.... 381
Bird's elements of natural philosophy 191
Bird, Dr. G. on the employment of elaterium ........................ 598
Bladder, inflammation of the. ....... 402
Bladder, Coulson on diseases of the.. 479
Bladder, spasm of the.............. 479
Blind catheter, Mr. Stilon's ........ 312
Blisters, M. Trousseau on .......... 547
Blood, detection of arsenic in the.... 226
Blood, Magendie on the alterations of 228
Bloodletting in infants ............ 414
Blue-pill and calomel, Dr. Sigmond on 417
Bones, arsenic in the .............. 542
Brain, gradual development of the.... 411
Brain, disease of, Mr. H. J. Johnson's case of.......................... 578
Breast, Cooper's anatomy of the .... 360
Breast, injection of the ............ 361
Breast, form and situation of the.... 362
Breast, structure of the............ 364
Breast, internal parts of the ........ 367
Breast, fat of the.................. 372

Breast, effects of gestation, &c. on the, 374
Bright on spasmodic disease ........ 2
Bright's elements of the practice of medicine ........................ 136
British reptiles .................. 1S2
British fishes, Yarrell's ............ 192
British birds, Yarrell's ............ 192
Brodie's, Sir B., retirement from St. George's Hospital ............... 602
Bronchial catarrh ................ 407
Bronchitis ....................... 407
Bronchitis, chronic............. .... 407
Bubo ............................ 157
Budd on the pathology of the spinal cord ......................... 30
Burne on tuphlo-enteritis .......... 43
Burne, Dr. on habitual constipation.. 467
Burnett's, Sir William, patent ...... 618
Burying-places of the Metropolis.... 59
Busk's case of aneurysmal tumor.... 22

C.

Cæcum, inflammation of the........ 534
Carcinoma uteri, Mr. Lever on...... 25
Carcinoma uteri, duration of........ 26
Carditis, Mr. Salter's case of........ 51
Carmichael, Mr. on the origin of malignant diseases ................. 594
Carotid artery, internal aneurysm of.. 575
Cataleptic ecstacy ................ 559
Catarrh ......................... 143
Cellular tissue, inflammation of the .. 535
Cerebritis ... ................... 146
Cerebro-spinal apparatus ........... 411
Cervix uteri, scarifying the ........ 620
Ceylon moss, Previte's prepared .... 600
Charenton lunatic asylum.......... 566
Children, incontinence of urine in .. 542
Chlorosis, Mr. Fox on ............ 161
Chloruretted injections, utility of .... 239
Cholera infantum ................ 399
Cholera, treatment of, with acetate of lead ........................... 593
Chronic hydrocephalus, pressure in .. 573
Civiale on the treatment of gravel .. 203
Civilization, Dr. Robertson on...... 169
Classification of infectious agents .. 451
Clergy, frightful state of the........ 285
Climate of Naples, Dr. Strange on the 307
Climate of New Zealand, Swainson on 445
Club-foot, Dr. Little on .......... 423
College of surgeons ............... 287
Comminuted fractures, advantages of delay in ....................... 216
Compound fractures of the cranium 571
Congenital tumor of the neck, Mr. Hawkins on .................... 7
Congenital absence of the pericardium 29
Congenital malformations.......... 411
Congestion of the liver............ 399
Congestive inflammation of the larynx 404
Constantinople lunatic asylum...... 569
Constipation, habitual, Dr. Burne on 467
Continued fever ................. 141
Convulsions .................... 413
Cooper on dislocation....... ...... 241
Cooper on amputation ............ 257
Cooper on hernia ................ 258
Cooper, Sir A. on the anatomy of the breast ........................ 360
Copeland's dictionary of practical medicine ...................... 450
Coracoid process, fracture of the .... 41
Corpus luteum, structure of the .... 54
Corrigan on acute rheumatism...... 266
Corrosive sublimate, treatment of malignant ulcers with.............. 526
Corrosive sublimate, poisoning by .. 615
Coryza ........................ 403
Costello's cyclopædia of surgery .... 450
Coulson on diseases of the bladder .. 479
Cranium, compound fractures of the 605
Croup...................... 143, 404
Cubebs should be fresh powdered for use ... ...................... 594
Cuichunchulli plant in elephantiasis.. 489
Curling on congenital absence of pericardium ...................... 29
Cutaneous diseases, Dr. Willis on.... 493
Cyclopædiæ of anatomy & physiology, 81
Cyclopædia of practical surgery .... 450
Cystitis .... ................... 145

D.

Davy, Sir Humphry, memoirs of .... 317
Death of Mr. Copland Hutchison.... 602
Deglutition, congestion of the organs of 392
Dextrine bandages for fractures .... 591
Diagnosis of stomach disorders...... 232
Dictionary of practical medicine ... 450
Digestive organs, diseases of the .... 390
Diseases of the heart, Dr. Hope on .. 73
Diseases of the eye, Mackenzie on .. 188
Diseases of infants............... 208
Diseases of infants, M. Billard on the 381
Diseases of infants................ 386
Diseases of the skin ..... ........ 387
Diseases of the digestive organs...... 390
Diseases arising from dentition...... 391
Diseases of the bladder, Mr. Coulson on 479
Diseases prevalent at Algiers........ 516
Dislocation of the patella outwards .. 278
Dislocation of the head of the radius backwards ..................... 285
Dislocations, Sir A. Cooper on...... 241
Dreams, theory of ................ 329
Drunkenness in France ............ 198
Dry gangrene, remarkable case of.... 28
Duration of human life............ 193
Duration of life, influence of sex on 193
Duration of generations ........... 193
Duration of human life ............ 550

E.

Earle on lunatic asyla ............ 563
East, state of medicine in the........ 273
Eczema of the scalp .............. 494

Edwards on animal heat .......... 94
Effects of lead upon the system....... 11
Elements of medicine, Gregory's .... 188
Elements of natural philosophy...... 191
Encysted tumors ................ 184
Encysted tumors of the eyelids...... 185
Encysted ganglionic swellings, treatment of ...................... 555
Enteritis ........................ 145
Enteritis, congenital .............. 397
Epidermis, exfoliation of the....... 384
Epilepsy, cured .................. 208
Epilepsy ........................ 549
Epilepsy after trephining .......... 572
Evidence of medical witnesses ...... 120
Excision of the tonsils ....... .... 187
Exfoliation of the epidermis........ 384
Eye, Mackenzie on diseases of the .. 188

F.

Femur, congenital luxation of the .. 413
Fenner on scarification of the cervix uteri ........................ 620
Fergusson on yellow fever.......... 297
Fever ........................... 137
Fever, idiopathic ................ 137
Fever, intermitting................ 137
Fever, remittent ................ 138
Fever, infantile remittent ... ...... 139
Fever, continued ................ 141
Fever hospital, report of the ...... 171
Fibrine, Gulliver on softening of ... 47
Fibrine, concoction of ............ 48
Fibrine, softened, in the heart and aorta ........................ 49
Fibrine softened, in the veins ...... 49
Fingers, tremor of the, in writing .. 518
Fleming, Dr. on abscess between the pharynx and spine ..... ........ 582
Flood's anatomy of the arteries .... 167
Fluoride of calcium, Dr. Rees on.... 251
Fœtal pulse...................... 513
Fœtus, uterine vagitus of the ...... 514
Foote on the oil of turpentine in iritis 487
Form and situation of the breast.... 362
Fox on chlorosis.................. 161
Fracture of the coracoid process of the scapula ..................... 41
Fractures, Sir A. Cooper on ........ 241
Fractures of the humerus.......... 244
Fractures, dextrine bandages for .... 591
France, drunkenness in............ 198
Frequency of herniæ at different ages 529
Frightful state of the clergy ........ 285

G.

Gangrene of the lungs ............ 607
Gastric indigestion................ 393
Gastritis ........................ 393
Gastro-hepatic irritation .. ....... 471
Gatherings from Grave Yards ...... 56
Gelatinous softening .............. 394
Gendrin's theory of menstruation .. 514
Genius, sons of .................. 322
Glanders, acute .................. 543
Glasgow lunatic asylum............ 557
Grave yards, Mr. Walker on ...... 56
Gravel, M. Civiale on ............ 203
Gregory, on small-pox ............ 17
Gregory's elements of medicine .... 188
Groin, Morton's anatomy of the.... 167
Guerin on spinal curvature .... ... 528
Gulliver on softening of fibrine .... 47
Gunshot wound of the arm ........ 608
Guy's hospital report.............. 241
Guy on the pulse in phthisis ...... 256

H.

Habitual constipation, Dr. Burne on, 467
Habitual constipation, neuralgia from 467
Hall on the nervous system ........ 36
Hall on hibernation .............. 81
Hanging, signs of death from ...... 203
Hanwell lunatic asylum .......... 563
Hawkins on congenital tumor of the neck ........................ 7
Head, injuries of the.............. 570
Heard on ununited fracture ........ 262
Heart, Dr. Hope on diseases of the.. 73
Heart, colour of the .............. 410
Hepatitis........................ 146
Hernia, Mr. Cooper on ............ 259
Hernia.......................... 401
Herniæ at different ages..... ...... 529
Herpes, treatment of............. 451
Hibernation, Dr. M. Hall on ...... 81
Hibernating animals, sleep of ...... 84
Hiccup, treatment of.............. 451
Hooping-cough ... .............. 408
Hope on diseases of the heart ...... 73
Hughes on incipient phthisis........ 252
Human body, temperature of the.... 96
Human life, duration of .......... 193
Human life, duration of .......... 550
Hutchison, Mr. Copland, death of .. 602
Hydrocele of the neck ............ 212
Hydrocephalus, congenital ........ 411
Hypertrophy danger of large blood-letting in............. ........ 451

I.

Icthyosis ...................... 499
Idiopathic fever .................. 137
Imperforate anus ................ 395
Impotent, out door relief of the...... 117
Incipient phthisis, Dr. Hughes on .. 252
Incontinence of urine in children.... 542
Infant brain, congestion of the...... 412
Infantile remittent fever .......... 139
Infantile remittent fever, Dr. M'Cabe on .......................... 617
Infants, diseases of................ 208
Infants, M. Billard on the diseases of 381
Infants, colic of ........ ........ 396
Infants, ophthalmia of ............ 414
Infants, viability of .............. 415
Infectious agents, classification of .. 451
Inflammation .................... 143

Inflammation of the mucous membranes .......................... 143
Inflammation of the serous membranes .......................... 145
Inflammation of the parenchyma of organs .......................... 146
Inflammation .......................... 412
Inflammation of the cæcum .......................... 534
Inflammation of the cæcum, acute .. 534
Influenza .......................... 143
Injuries of the head .......................... 570
Inoculation of syphilitic diseases .... 155
Insane, suicide among the .......... 557
Insane, mortality of the .......... 557
Insane, diet of the .......................... 560
Insanity, Dr. Millingen on .......... 507
Intermitting fever .......................... 137
Intestines, spasm of the .......... 397
Introductory lecture, Mr. H. J. Johnson's .......................... 289
Iritis, oil of turpentine in .......... 487
Itinerant phrenologists .......................... 282

J.

Jaundice of infants .......................... 400
Johnson's, Mr. H. J. introductory lecture .......................... 289
Johnson's, Mr. H. case of disease of the brain .......................... 578
Jones's animal kingdom .......... 192
Judd's preparation of the piper cubeba 486

K.

Kennedy's medical report .......... 171

L.

La Salpêtrière .......................... 566
Labouring classes, medical relief for .. 113
Lactation, on .......................... 376
Language, phrenological organ of .... 519
Larynx, congestion and inflammation of 404
Le Bicêtre .......................... 566
Lead, effects of, upon the system .... 11
Lead, paralysis from the absorption of 12
Lead, acetate of, in cholera .......... 593
Lee on the structure of the corpus luteum .......................... 54
Lee on nervous disorders .......... 577
Legal medicine, microscope in ...... 201
Lepra vulgaris .......................... 493
Lever on carcinoma uteri .......... 25
Limerick infirmary .......................... 605
Liston on anasarcous tumor of the scrotum .......................... 19
Liston's practical surgery .......... 178
Little on club-foot .......................... 423
Lizar's anatomical text-book .......... 167
Lumbrici discharged from an umbilical abscess .......................... 219
Lunatic Asylum, Glasgow, report from 557
Lunatic Asylum, York, report from 562
Lunatic asylums, Dr. Earle on ...... 563
Lunatic asyla in Europe, account of, 563
Lungs, congestion of the .......... 406
Lungs, œdema of the .......... ... 408
Lungs, gangrene of the .......................... 607
Luxation of the femur, congenital .. 413
Lymphatic system .......................... 414
Lynn's, Dr. case .......................... 618

M.

M'Cabe on infantile remittent fever . 617
Mackenzie on diseases of the eye .... 188
Malcolmson on sloughing of abdominal parietes .......................... 262
Malignant disease of the tongue .... 14
Malignant ulcers, treatment of ...... 526
Malignant diseases, Mr. Carmichael on the origin of .......................... 594
Mamma, injection of the .......... 361
Mamma, form and situation of the .. 362
Malta, lunatic asylum at .......... 569
Maygrier's anatomist's manual ...... 167
Mayo on syphilis .......................... 504
Measles, Mr. Webster on .......... 27
Medical etiquette, Mr. Banks on .... 62
Medical certificates, remuneration for 69
Medical relief for the working classes 113
Medical witnesses, evidence of ...... 130
Medical school at Athens .......... 275
Medical biography .......................... 317
Medicine, Bright and Addison's practice of .......................... 136
Medicine, Gregory's elements of .... 188
Medicine, state of, in the East ..... 273
Medicine, Copeland's dictionary of .. 450
Medico-Chirurgical Transactions .... 1
Medico-parochial attendance, Yeatman on .......................... 113
Medico-botanical society, transactions of the .......................... 486
Memoirs of Sir Humphry Davy .... 317
Memoirs of celebrated physicians and surgeons .......................... 317
Memoirs of Dr. Physick .......... 317
Menstruation, Gendrin's theory of .. 515
Mercury, preparations of .......... 151
Mercury, cyanuret of .......................... 152
Mercury, blue pill and calomel, Dr. Sigmond on .......................... 417
Microscope in legal medicine .......... 201
Milan, lunatic asylum at .......... 568
Milk, influence of medicines on .... 380
Milky urine, case of .......................... 556
Millingen on insanity .......................... 507
Mitral valve, the pulse in disease of the 74
Montyon prizes .......................... 602
Morton's anatomy of the groin ...... 167
Mott on the state of medicine in the East .......................... 273
Mouth, epidemic apthous inflammation of .......................... 533
Mucous membranes, inflammation of 143
Muguet of the mouth .......................... 390
Muriate of gold .......................... 154
Muscular irritability in paralytic limbs 36

N.

Naples, Dr. Strange on the climate of, 307

Natural philosophy, Bird's elements of 191
Neck, Hawkins on congenital tumor of the ........ 7
Neck, hydrocele of the ........ 212
Nephritis ........ 148
Nervous system, Dr. Hall on the .... 36
Nervous disorders, Mr. Lee on ...... 577
Neuralgia from habitual constipation, 469
New noses ........ 183
New Zealand, Swainson on climate of, 445
Nostrils, foreign bodies in the ...... 186

O.

Obliteration of the fœtal openings ... 409
Obstetric plates, Dr. Ramsbotham's.. 508
Obstetric auscultation ........ 509
Occurrences at the small-pox hospital 17
Œdema of the lungs ........ 409
Œsophagus, foreign bodies in the.... 187
Operation for artificial anus ........ 539
Operations, treatment of wounds after 215
Operators and operations........ 283
Ophthalmia of infants ........ 414
Orbit, aneurysmal tumor of the...... 22
Orfila on signs of death from hanging, 203
Organs of locomotion........ 413
Osseous transformation ........ 465
Out-door relief of the impotent ..... 117
Oxydes of iron as antidotes of arsenic, 225

P.

Paralysis from the absorption of lead, 12
Paralytic limbs, muscular irritability in 36
Paralytic limbs, influence of emotion in 39
Paralytic limbs, action of strychnine on 40
Parenchyma of organs, inflammation of 146
Parker on syphilis ........ 149
Parochial medical relief........ 113
Patella, dislocation of the, outwards.. 279
Pathology of the spinal cord. By Dr. Budd ........ 30
Pathology of tetanus ........ 35
Pathology of the veins in the abdomen 524
Penis; amputation of the........ 532
Pennsylvania hospital ........ 570
Pericarditis ........ 146
Pericardium, congenital absence of the 29
Pettigrew's medical portrait gallery.. 317
Phrenitis ........ 146
Phrenological organ of language ..... 519
Phthisis ........ 146
Phthisis, Guy on the pulse in ...... 256
Phymosis, barbarous operation for .. 156
Physick, Dr. life of........ 317
Physick, Dr. anecdote of ........ 353
Piper cubebæ, new preparation of ... 486
Pleuritis ........ 146
Plugging the posterior nares........ 186
Pneumonia ........ 146
Pneumonia ........ 407
Poisoning by arsenic, treatment of .. 220
Poisoning from the extract of aconite, 239
Poisoning by sulphuric acid ........ 250
Poisoning by corrosive sublimate.... 615
Pons varolii, apoplexy of the........ 33
Poor Law Amendment Act ........ 113
Poor Law Commissioners, reports of.. 113
Porrigo scutulata ........ 494
Porter on the cuichunchulli plant.... 489
Poverty, its evils........ 113
Practical surgery, Liston's........ 178
Practical medicine, dictionary of .... 450
Practical surgery, cyclopædia of .... 450
Practice of medicine, elements of the, 136
Preservation of anatomical subjects.. 248
Prosecution of a patient by her doctor 523
Prostate gland, Coulson on diseases of 479
Prostate gland, affections of the. .... 482
Prostate gland, acute inflammation of 482
Prostate gland, chronic inflammation of 483
Prussic acid, therapeutical properties of 549
Psoriasis annularis........ 497
Pulse, state of the, in children ...... 385
Purpura, case of........ 614

R.

Radius, dislocation of the head of the, 285
Ramsbotham's obstetric plates...... 508
Randolph's life of Dr. Physick ...... 317
Rees on fluoride of calcium ........ 251
Regulations respecting assistant-surgeons in the Navy ........ 601
Reid's cases ........ 613
Remittent fever ........ 138
Remuneration for medical certificates, 69
Report of the fever hospital ........ 171
Report from the York lunatic asylum, 562
Reports of the poor-law commissioners 113
Respiration, imperfect establishment of 406
Retroversion of the uterus, Mr. Halpin on ........ 574
Revivescence ........ 92
Rheumatism of the uterus........ 238
Ricord on the treatment of syphilis .. 235
Robertson on civilization ........ 169
Robertson on the teeth ........ 490
Rumsey on medico-parochial attendance ........ 113
Rumsey, letter from Serj. Talfourd to, 113
Rupture of the axillary artery ...... 267
Rupture of the aorta ........ 612

S.

Salter's case of carditis ........ 51
Scabies ........ 495
Scapula, fracture of the coracoid process of the ........ 41
Scarifying the cervix uteri........ 620
Schneiderian membrane, inflammation of the ........ 578
Scrotum, anasarcous tumor of the ... 19
Self-supporting dispensaries ........ 113
Sigmond on tea ........ 132
Sigmond on mercury, blue-pill, and calomel ........ 417
Sketches of French surgery & surgeons 586
Skin in infants, colour of the ....... 382
Sloughing of the abdominal parietes.. 262

Sloughing of a brachial aneurysm.... 281
Sloughing of the uvula from catarrh.. 586
Small-pox hospital, occurrences at the 17
Small-pox, Dr. Gregory on ......... 17
Solly's case of dry gangrene ........ 28
Sons of genius .................... 322
South on fracture of coracoid process 41
Spain, state of the profession in .... 592
Spasm of the bladder .............. 479
Spasmodic disease, Dr. Bright on.... 2
Spinal cord, Dr. Budd on the pathology of the .................... 30
Spinal curvature, M. Guerin on .... 528
Starched bandage, use of the........ 527
Stercoral inflammation of the ...... 538
Sterno-mastoid, removal of the ..... 182
Stewart on the diseases of infants.... 381
Stilon on the blind catheter ........ 312
Stomach disorders, diagnosis of...... 232
Strange on the climate of Naples .... 307
Structure of the corpus luteum...... 54
Strychnine, action of, on paralytic limbs ........................ 40
Subjects, supply of................. 286
Sulphuric acid, poisoning by........ 250
Supply of subjects ................. 286
Surgeons, new regulations of the College of........................ 287
Swainson on the climate of N. Zealand 445
Syphilis, Mr. Parker on............ 149
Syphilis, Ricord on the treatment of.. 235
Syphilis, Mr. Mayo on ............ 504
Syphilitic diseases, inoculation of.... 155
Syphilitic eruptions ............... 387

T.

Talfourd's letter on the poor-laws.... 113
Talipes equinus, congenital ......... 425
Talipes equinus, non-congenital .... 426
Talipes tarsus ........... ........ 426
Talipes varus ..................... 427
Talipes valgus.................... 428
Talipes calcaneus ................. 436
Talipes, treatment of ........... .. 437
Tea, Dr. Sigmond on............... 132
Teeth, Robertson on the............ 490
Temperature of the human body .... 94
Temperature, influence of, on vitality, 111
Tetanus, pathology of........... .. 35
Tetanus ......................... 413
Theory of dreams ................. 329
Therapeutical properties of blisters .. 549
Timber preservative company ...... 618
Todd's Cyclopædia of Anatomy...... 81
Tongue, malignant disease of the .... 14
Tonsils, excision of the ............ 186
Torpor from cold .................. 93
Transactions of the Medico-botanical Society......................... 486
Triceps, rupture of the tendon of the, 220
Trichosis ......................... 496
Trousseau on blisters... .......... 547
Tubercular disease, Dr. Gerhard on .. 590
Tumor-follicularis .......... ..... 503
Tumors, encysted ................. 185
Tuphlo-enteritis, Dr. Burne on...... 43
Tuphlo-enteritis, analysis of cases of.. 46
Turpentine, oil of, in iritis.......... 487
Typhus .......................... 456

U.

Umbilical abscess, lumbrici discharged from ........................... 219
Umbilical cord, separation of the .... 382
Union workhouses................. 456
Ununited fractures, Dr. Heard on.... 262
Urinary apparatus, malformations of, 402
Urine, retention of................ 402
Uterine vagitus of the fœtus ........ 514
Uterus, rheumatism of the ......... 238
Uterus, retroversion of ............ 574
Utrecht lunatic asylum ............ 565
Uvula, sloughing of, from catarrh.... 586

V.

Vaccination...................... 600
Vagina, complete absence of the .... 531
Valgus........................... 428
Valves, disease of the.............. 74
Varicose veins, twisted suture for.... 272
Veins in the abdomen, pathology of.. 524
Venereal, the ..................... 149
Venice, lunatic asylum at.......... 568
Viability of infants................ 416
Vitality, influence of temperature on, 111
Vitiligo .......................... 498
Vulva, gangrene of the ............ 400

W.

Wakefield lunatic asylum .......... 563
Walker on grave-yards ............ 56
Watson's clinical report............ 270
Weale's case of rupture of the aorta.. 612
Webster on measles............... 27
Wounds, dressing of.............. 179
Wounds, treatment of, after operations 215
Willis on cutaneous diseases........ 493
Writing, tremor of the fingers in .... 518

Y.

Yarrell's history of British birds .... 192
Yarrell's history of British fishes .... 192
Yeatman on medico-parochial attendance .......................... 113
Yellow fever, Dr. Fergusson on ..... 297
York lunatic asylum, report from, 562, 564

### BIBLIOGRAPHICAL RECORD.

1. State of an Institution near York, called the "Retreat," for Persons afflicted with Disorder of the Mind. York, 1839.

2. Medical Portrait Gallery, Part 20. Price 3s. By Mr. Pettigrew. 1. Memoir and Portrait of Harvey. 2. Ditto of Dr. Marshall Hall.

3. The Phrenological Journal, Quarterly. By Hewett Cottrell Watson, Esq. No. VIII. New Series. Oct. 1839.

4. Notice sur le Monesia. Paris, 1839.

5. Cases of Chronic Hydrocephalus, or Water in the Head; with Observations, and a Detail of a new and successful Plan of Cure. By J. E. Barnard, M.R.C.S. &c. Octavo, pp. 30. London, 1839.

6. Medical Miscellany, October, 1839, No. 1. Price 1s. 6d. Monthly. Sherwood and Co.

7. The Cyclopædia of Anatomy and Physiology. By Dr. Todd. Part XVIII. Containing Insects—Insectivora. Illustrated with numerous Engravings. Sherwood and Co. 1839.

8. Londres Ancien et Moderne; ou Recherches sur l'Etat Physique et Social de cette Metropole. Par A. M. Bureaud-Riofrey, M.D. Octavo, pp. 136. Londres et Paris, Chez Bailliere, 1839.

9. Elements of the Theory and Practice of Medicine, designed for the Use of Students and Junior Practitioners. By George Gregory, M.D. Fellow of the Royal College of Physicians, &c. Octavo, pp. 774. Renshaw, Strand; Churchill, Princes-street. Fifth Edition, revised and greatly enlarged.

10. The Medical Examiner. Edited by J. B. Biddle, M.D. and W. W. Gerhard, M.D. Published weekly, price 5 dollars per annum. *In exchange.*

☞ *We have received through the kindness of the editors, the numbers of this valuable contemporary up to No.* 41, *Oct.* 12*th*, 1839.

11. The Medical Times—a Weekly Journal of English and Foreign Medicine and Medical Affairs. Nos. 1 to 13. Price 3d. 421, Oxford Street. *In Exchange.*

12. The Physiognomy of Mental Diseases. By Sir A. Morison, M.D. No. 15. Price 3s. 6d. Dementia.

13. The Anatomist's Manual; or a Treatise on the Manner of preparing all the Parts of Anatomy, &c. By J. P. Maygrier, M.D. &c. Translated from the last French Edition. Octavo, pp. 560. George Henderson, London, 1839.

14. Text-Book of Anatomy for Junior Students. By Alex. Jardine Lizars, M.D. Part I. Pages 272. Bell and Bradfute. Edinb. 1839.

15. On the Practical Use of Inhalations in Diseases of the Throat and Chest. By John Harwood, M.D. F.R.S. &c. Physician to the Dispensary of St. Leonard's. Octavo, pp. 68. Second Edition. Hatchard and Sons, 1839.

16. Clinical Remarks on some Cases of Liver Abscess presenting externally. By J. G. Malcolmson, M.D.

17. Guy's Hospital Reports, No. 9, Oct. 1839. Edited by G. H. Barlow, M.A. Trin. Col. Camb.; and J. P. Babington, M.A. Trin. Col. Camb. S. Highley, London. 1839.

18. The Modern Treatment of Syphilitic Diseases, both primary and secondary, comprising an Account of the New Remedies, with numerous Formulæ for their Preparation and Administration. By Langston Parker, Esq. M.R.C.S. Lecturer on Anatomy, &c. Octavo, pp. 158. Churchill, London. October, 1839.

19. Valedictory Address to the Students in Medicine of the College of Physicians and Surgeons, New York. By J. B. Beck, M.D. New York, 1839.

☞ *Excellent advice in affectionate terms.*

20. The New York Journal of Medicine and Surgery. Published Quarterly. No. 2.

Oct. 1839. Churchill, London. *In exchange.*

21. Affinities of Plants; with some Observations upon progressive Development, &c. By THOMAS BASKERVILLE, M.R.C.S. London, Taylor and Walton. Octavo, pp. 114. 1839.

22. Tea: its Effects, Medicinal and Moral. By G. G. SIGMOND, M.D., &c. Octavo, pp. 144. Longman and Co. 1839.

23. A Series of Anatomical Plates. By JONES QUAIN, M.D. Fas. 75. Price 2s. Taylor and Walton. Oct. 1839.

24. A Practical Treatise on Diseases of the Eye. By WILLIAM MACKENZIE, M.D. Surgeon-Oculist in Ordinary to Her Majesty, in Scotland; Lecturer on the Eye in the University of Glasgow, &c. To which is prefixed, an Anatomical Introduction explanatory of a Horizontal Section of the Human Eye-ball. Third Edition, pp. 933. Glasgow.

25. The Eye; a Treatise on the Art of preserving this Organ in a Healthy Condition, and of improving the Sight, &c. &c. with Observations on the Expression of the Eye as indicative of the Character and Emotions of the Mind. By J. CH. AUGUST. FRANZ, M.D. &c. 8vo, pp. 296. Churchill, London, Nov. 1839.

26. Obstetric Auscultation. By Dr. H. F. NAEGLE, M.D. Translated from the German, by CHARLES WEST, M.D. Graduate of the University of Berlin. Small 8vo. pp. 120. Renshaw, Nov. 1839.

27. The enlisting, discharging, and pensioning of Soldiers, with the Official Documents on these Branches of Military Service. By HENRY MARSHALL, F.R.S. Deputy Inspector of Army Hospitals. Second Edition. Edinburgh, A. & C. Black. London, Longman & Co. 1839.

28. A Treatise on the Medical Jurisprudence of Insanity. By J. RAY, M.D. With an Introductory Essay. By D. SPILLAN, M.D. Octavo, pp. 436. G. Henderson, Old Bailey. Nov. 1839.

29. Elements of Physiology. By J. MÜLLER, M.D. Translated from the German, by WILLIAM BALY, M.D, Illustrated with Steel Plates and Wood Engravings. Second Edition. Part I. General Physiology. Taylor and Walton. Nov. 1839.

30. Remarkable Case of the Effects of Lightning on the Human Body, with Observations on the Nature and Phenomena of Lightning, adapted for general Readers. By JOHN DAVIES, Surg. to the General Infirmary at Hertford. Price 1s. 1839.

31. West's New Dissecting or Surgical Microscope, mounted in Brass, German Silver, and Silver. Price 8s. 6d., 12s. 6d., or 21s.

☞ *A very useful Instrument for examining diseases of the skin and minute structures, anatomical or pathological.*

32. Surgical and Medical Cases, reprinted from the Medico-Chirurgical Journals, with Observations. By W. BRIGHAM, M.R.C.S. Highley, Fleet Street. 1839.

33. A Lecture introductory to the Business of the original School of Medicine. Peter Street, Dublin. By W. TAGERT, M.R.C.S. Dublin, Nov. 1839.

34. Observations on Yaws, and its Influence in originating Leprosy, &c. By JAMES MAXWELL, M.D., formerly Surgeon of the Annota Bay Hospital, Jamaica. 8vo, pp. 134. Maclachlan, Stewart, and Co. Edinburgh. Nov. 1839. With Plates.

35. The India Journal of Medical and Physical Science, &c. for March, April, and May, 1839; also the India Review for the same months. *In exchange.*

36. A Treatise on the Diseases of Infants, founded on recent Clinical Observations and Investigations, &c. By C. M. BILLARD. Translated from the Third French Edition. By JAMES STEWART, M.D.

37. The American Phrenological Journal, No. 11. By Dr. CALDWELL. Philadelphia, Aug. 1839.

38. Gazette des Medecins Praticiens. Publié par le Docteur Amedée Latour. No. 41. Published every fifth day. Single Sheet. Paris.

39. Gatherings from Grave Yards; particularly those of London: with a concise History of the Modes of Interment among different Nations, from the earliest Periods; and a Detail of Dangerous and Fatal Results produced by the unwise and revolting Custom of inhuming the Dead in the midst of the Living. By G. A. WALKER, Surgeon. Octavo, pp. 258. Longman & Co. November, 1839.

40. Elements of Natural Philosophy; being an experimental Introduction to the Study of the Physical Sciences. By GOLDING BIRD, M.D. F.L.S. &c. Physician to the Finsbury Dispensary, and Lecturer on Natural Philosophy at Guy's Hospital. 8vo, pp. 407. Churchill. Nov. 1839.

☞ *An admirable manual, not merely for medical students, but for medical men of every description.*

---

41. The Physiognomy of Mental Diseases. By ALEXANDER MORISON, M.D. No. 16. Idiocy. Price 3s. 6d.

---

42. A Lecture introductory to the Course of Lectures on the Principles and Practice of Medicine, delivered at the Aldersgate Street School, at the Commencement of Session 1839-40. By ROBERT WILLIS, M.D. Sherwood and Co. 1839.

☞ *A very able discourse, and such as might be expected from the talented lecturer.*

---

43. Medical Report of the House of Recovery and Fever Hospital, Cork Street, Dublin; for two Years, from Jan. 1837, to Dec. 1838. By G. A. KENNEDY, M.D. Dublin. Nov. 1839.

---

44. Transactions of the Royal Medico-Botanical Society of London, 32, Sackville Street. Octavo, pp. 248. Highley, Fleet Street, & Churchill, Princes St. Nov. 1839.

---

45. The Medical Service in India. A Letter from a "Bengal Surgeon."

☞ *We regret that we cannot print this paper (it is indeed already printed) according to the wish of the writer, as it would occupy* 20 *pages or more of the Journal. We shall endeavour to notice it in our next number.*

---

46. Read's Resuscitator, for restoring Persons in cases of Suspended Animation, from Drowning, Suffocation, Strangulation, Noxious Vapours, Cold, &c. With Apparatus for furnishing a Warm-Air Bath or Vapour-Bath. Manufactured and sold by John Reid, 35, Regent Circus.

☞ *We have seen this Apparatus, which is very ingenious, as are all Mr. Reid's instruments.*

---

47. The Medical Jurisprudeuce of Insanity. By J. M. PAGAN, M.D. Lecturer on Medical Jurisprudence, &c. Glasgow, 8vo, pp. 330. London, Ball and Arnold—Glasgow, John Symington. Nov. 1839.

48. Elements of Surgery. By ROBERT LISTON, Surgeon to the North London Hospital, Professor of Clinical Surgery, &c. Second Edition, greatly enlarged. Longman and Co. 1840.

---

49. A new Treatment of Anchylosis. By Dr. REA BARTON. (From the North American Medical and Surgical Journal.)

---

50. Clinical Report from the Wells Hospital for the Blind and Lame. By Dr. ISAAC HAYS.

---

51. The final Report of the Committee of the Philadelphia Medical Society, on the Construction of Instruments for the Radical Cure of Hernia, &c. By ROBERT CHASE, M.D. Philadelphia, 1837.

---

52. Memoir of the Life and Character of PHILIP LYNG PHYSICK, M.D. By JOHN RANDOLPH, M.D. Philad. 1839.

---

53. First Lines of Physiology, designed for the Use of Students in Medicine. By DANIEL OLIVER, M.D. Boston, 1835.

---

54. The Naturalist's Library. Conducted by Sir W. JARDINE, Bart. Mammalia. Vol. IX. Dogs. Including also the Genera Hyæna and Proteles. By Lieut.-Colonel CHARLES HAMILTON SMITH. Edinburgh, Lizars. London, Highley, 1839.

---

55. Medical Etiquette; or an Essay upon the Laws and Regulations which ought to govern the Conduct of Members of the Medical Profession in their Relation to each other. Compiled exclusively for the Profession. By ABR. BANKS, Esq. M.R.C.S. Small 8vo, pp. 104. Fox, Paternoster Row. Dec. 1839.

---

56. A Compendium of Materia Medica, Pharmacy, and Toxicology; containing Tables of the Materia Medica and Chemical Decompositions, &c. &c. for the Use of Medical Students. By D. SPILLAN, M.D. Duodecimo, price 2s. G. Henderson, Old Bailey. Dec. 1839.

---

57. Observations on Medical Education, with a View to Legislative Interference. By RICHARD JONES, M.R.C.S. and late of the Hon. East India Company's Service. Octavo, pp. 52. Dedicated, by permission, to Sir Robert Peel, Bart. Murray, Albemarle Street. Dec. 1839.

